# Barriers to accessing psychotherapeutic care among young adults: Individual and regional associated factors

**DOI:** 10.25646/13549

**Published:** 2025-11-19

**Authors:** Vera Birgel, Paul Gellert, Heike Hölling, Ronny Kuhnert, Niels Michalski, Michael Rapp, Julie L. O’Sullivan, Caroline Cohrdes

**Affiliations:** 1 Robert Koch Institute, Department of Epidemiology and Health Monitoring, Berlin, Germany; 2 German Centre for Mental Health, Berlin/Potsdam, Germany; 3 Charité – Universitätsmedizin Berlin, Institute for Medical Sociology and Rehabilitation Science, Berlin, Germany; 4 University of Potsdam, Chair of Social and Preventive Medicine, Potsdam, Germany

**Keywords:** Young adults, Health-related quality of life, Health literacy, Help-seeking behaviour, Social status, Mental health, Anxiety, Regression analysis

## Abstract

**Background:**

Mental health problems are widespread among young adults in Germany, yet the use of professional help remains low. This study aimed to identify the most common perceived barriers to help-seeking and to examine which individual and regional factors are associated with unmet need for mental health care.

**Methods:**

A total of 3,051 former participants of the KiGGS cohort study (aged 16 to 25 years) took part in an online survey. Group comparisons and regression analyses were conducted to examine associations of individual and regional characteristics with unmet need for care.

**Results:**

Overall, 42.6 % of respondents reported not having used professional help despite being advised to do so or perceiving a need themselves. The most frequently reported barriers were difficulties in acknowledging one’s own need for help (problem denial) and negative attitudes towards seeking professional help (help-seeking stigma). Within this group, unmet need was more likely reported by women, individuals with lower subjective social status, depression or anxiety symptoms, low mental health-related quality of life, or inadequate health literacy. Regional characteristics such as the density of care provision or socioeconomic deprivation were not significantly associated with unmet need.

**Conclusions:**

The findings highlight the importance of subjective barriers to help-seeking behaviour. Low-threshold interventions may help improve self-awareness, appraisal competence, and openness in dealing with psychological distress, thereby reducing the risk of mental health disorders.

## 1. Introduction

Mental health disorders are among the leading causes of disease burden worldwide, particularly in young adults [1]. Approximately 75 % of all severe mental disorders manifest before the age of 25, underscoring the importance of early detection and access to care [2]. During this developmental stage, tasks such as gaining autonomy, forming identity, and transitioning into education or employment may increase vulnerability to psychological distress [3]. Early access to preventive and therapeutic services [4], awareness of one’s own mental health, readiness to seek help, and knowledge of available support options, are crucial to prevent long-term impairments [5].

Epidemiological studies indicate that psychopathological symptoms and mental disorders have increased both in Germany and internationally, particularly among young people [6–9]. During the COVID-19 pandemic, for instance, the prevalence of mental health problems among children and adolescents in Germany nearly doubled from 17.6 % to 30.4 % [10]. Although psychotherapy is considered as one of the most effective forms of treatment [11], actual service utilisation remains low. In a nationwide German survey, 12.9 % of adults reported having experienced emotional distress in the past three years that would have warranted medical or psychological help, yet more than a third did not seek psychotherapeutic care [12]. Moreover, unmet need for care has been linked to an increased risk of suicidal ideation and attempts, emphasising the public health relevance of this treatment gap [13]. Utilisation also varies by gender and age: women are more likely than men to seek psychotherapeutic services [12], while younger age at onset is associated with a lower likelihood of seeking professional help [14, 15].

**JEPSY**

Study on Mental Health in Emerging Adulthood

**Data holder:** Robert Koch Institute

**Objectives:** To examine the mental health, well-being, resources, and care needs of young adults in Germany, taking into account past data from childhood and adolescence collected in the ‘German Health Interview and Examination Survey for Children and Adolescents’ (KiGGS).

**Study design:** Cross-sectional survey with retrospective longitudinal linkage

**Population:** Individuals aged 16 to 25 who participated in KiGGS Wave 2 (2014 – 2017)

**Sampling:** All KiGGS Wave 2 participants aged 16 and older who had consented to re-contact were invited to participate

**Sample size:** 3,063 respondents

**Study period:** March to July 2024

**Survey waves:**

► KiGGS Wave 2 (2014 – 2017)

Further information in German is available at https://www.rki.de/DE/Themen/Nichtuebertragbare-Krankheiten/Studien-und-Surveillance/Studien/JEPSY-Studie.html

Help-seeking behaviour among young adults is influenced by a variety of individual, social, structural, and regional barriers. Key barriers include negative attitudes towards psychotherapeutic care (e. g., internalized help-seeking stigma), fear of public stigma, and a lack of information about available professional services. Studies from the United States and Australia have also shown that financial barriers and the absence of insurance are important in these contexts [15–20]. In addition, individual health literacy – that is, the ability to access, understand, appraise, and apply health-related information – may play a crucial role. More than half of the German population (54.3 %) demonstrate problematic or inadequate health literacy and report considerable difficulties in dealing with health-related information [21]. Among young adults, this applies to almost two-thirds [22]. For this age group, which is often still learning to navigate the health care system, this factor may be particularly relevant. However, only a few international studies to date have examined the impact of health literacy on help-seeking behaviour in this age group [23].

**Key messages**

► A substantial proportion of young adults with mental health problems do not seek professional help despite perceiving a need or having been advised to seek support.
► The most frequently reported barriers include problem denial (doubts about one’s own need for help) and internalised help-seeking stigma (negative attitudes towards accessing professional care).
► Unmet need for mental health care is more frequently reported by individuals with mental health problems (e. g. depressive or anxiety symptoms, reduced mental health-related quality of life), as well as by women, individuals with lower subjective social status, or those with limited health literacy.
► Mental health service density shows no significant association with unmet need.
► Access to professional mental health care should be improved through low-threshold prevention strategies, including the promotion of health literacy, destigmatisation efforts, and targeted mental health education.

Beyond individual factors, regional differences in the density of care provision may also play an important role. In Germany, the availability of psychotherapeutic care varies depending on geographical and socioeconomic conditions [24], with urban regions tending to have a higher density of psychotherapists than rural areas [25]. Empirical findings indicates longer waiting times and poorer service provision in less densely populated areas, resulting in regionally unequal access to care [26]. Regional deprivation – that is, socioeconomic disadvantage within a given area – can also affect mental health and access to care, for example through higher psychosocial stress, lower social support, or limited health infrastructure [27, 28].

The aim of the present study was to examine the frequency and type of barriers to accessing professional mental health care among young adults in Germany. Furthermore, differences between participants with and without indications of elevated need for care were examined, considering potential individual factors (e. g., age, gender, health literacy) and regional characteristics (e. g., service density, socioeconomic deprivation). The findings aim to identify barriers to seeking professional help, improve understanding of existing needs, and highlight starting points for preventive measures.

## 2. Methods

### 2.1 Sample and recruitment

The study on Mental Health in Emerging Adulthood (‘Studie zur psychsichen Gesundheit von jungen Erwachsenen in Deutschland’, JEPSY) is based on data from an online survey of individuals aged 16 to 25. The study was approved by the Ethics Committee of the German Psychological Society (DGPs) (2024-03-14WV).

Participants were recruited from the second wave of the German Health Interview and Examination Survey for Children and Adolescents (KiGGS, 2014 – 2017), conducted by the Robert Koch Institute [29]. A total of 11,737 former KiGGS participants were contacted. Participants were eligible if they were aged between 16 and 25 at the start of data collection (1 March 2024) and had consented to further participation. In total, 3,063 participants completed the online questionnaire. After excluding 12 participants due to implausible response patterns [30, 31], the final analytic sample comprised 3,051 respondents (68.3 % female; mean age: 22 years, SD ± 2).

### 2.2 Data

Unmet need for professional help was assessed following a series of questions on past mental health disorders, using the item: *‘Has it ever happened, once or more often, that you were advised to seek professional help or thought about it yourself, but did not do so?’* Respondents who answered ‘yes’ subsequently completed eight items from the ‘Barriers to Seeking Psychotherapy Scale’ (BAPS) [32], additional items from the Composite International Diagnostic Interview (CIDI-5) [33], and one open-ended response option (‘Other’). All items were rated on a six-point scale (1 = not at all true, 6 = completely true), with higher values indicating stronger perception of the respective barrier. According to the scoring manual, items were grouped into five categories: 1) fear of public stigma, 2) fear of the psychotherapeutic setting, 3) problem denial, 4) internalized help-seeking stigma, and 5) practical implementation/organisation. The individual items and their corresponding categories are shown in Table 1. The selection of instruments and items followed recommendations by a commission of the German Centre for Mental Health (DZPG) for the development of a Minimum Data Set (MDS) on mental health assessment [34]. Specific barriers were assessed only among participants reporting unmet need; therefore, all analyses on perceived barriers (including regression models) refer exclusively to this subgroup. Indicators of potential need for professional help included the following measures:

► Depressive and anxiety symptoms: Patient Health Questionnaire-4 (PHQ-4); 4 items rated 0 – 3. A positive screening (i. e. relevant symptom burden) was defined as PHQ-2 or GAD-2 ≥ 3 [35].
► Mental health-related quality of life (HRQoL): Two-item version of the PROMIS Global Mental Health Scale, categorised based on T-scores; for group-comparisons dichotomised analytically into *poor/fair* vs. *good/very good/excellent* (instrument-based 5-level response) [36].
► Substance use: DSM-5 Cross-Cutting Symptom Measure [37]; classified as elevated when reporting ‘four or more alcoholic drinks’ or ‘non-prescribed substances’ on more than half of the days.

Health literacy was assessed using three items (four-point Likert scale) capturing difficulties in accessing, understanding and applying health-related information. Classification into categories (‘inadequate’, ‘problematic’, ‘sufficient’, ‘excellent’) followed recommendations from the M-POHL network guidelines (2021) [38].

The sociodemographic variables examined included sex assigned at birth, age, and subjective social status (10-point scale adapted from the MacArthur Scale of Subjective Social Status, 1 = lowest to 10 = highest status) [39].

Regional indicators at the district level included:

► Socioeconomic deprivation: German Index of Socioeconomic Deprivation (GISD; quintiles). The GISD measures relative socioeconomic disadvantage across three dimensions of inequality: education, employment, and income [40],
► District type: Classified according to Eurostat (urban, intermediate, rural) [41],
► Mental health service density: Number of licensed medical and psychological psychotherapists per 10,000 inhabitants, based on data from the National Association of Statutory Health Insurance Physicians [25].

### 2.3 Statistical methods

All analyses were conducted using R version 4.4.2, applying survey weights to adjust for selective participation (for further details, see [42]). The weighting adjusts the sample to the distribution of participants from the original representative KiGGS study wave 2.

Descriptive statistics were used to describe the perceived barriers to accessing professional help. Group differences in perceived barriers were examined using Mann-Whitney U tests, comparing individuals with and without positive screening for depressive symptoms, anxiety symptoms, or problematic substance use, as well as between groups with differing levels of mental health-related quality of life. For this purpose, the five categories of the PROMIS Global Mental Health Scale were collapsed into two groups: ‘poor’/’fair’ and ‘good’/’very good’/’excellent’. These group comparisons were additionally stratified by sex to identify potential differences in barrier perception between women and men.

To examine associations between individual and regional factors and the likelihood of reporting unmet need, multilevel logistic regression analyses (mixed-effects models) with random intercepts at the district level were conducted, using the ‘survey’ package [43]. The intraclass correlation coefficient (ICC) was calculated to estimate the proportion of variance attributable to the contextual (district) level. Results are reported as odds ratios (ORs). Sex-stratified regression models were not performed due to insufficient statistical power within the respective subgroups.

## 3. Results

Overall, 42.6 % of respondents (n = 1,301) reported that they had not sought professional help despite having been advised to do so or perceiving a need themselves.

The highest levels of agreement among the assessed barriers were observed in the domains of problem denial (e. g., ‘I thought my problems were not severe enough to seek psychotherapy’) and internalised help-seeking stigma (e. g., ‘I thought I had to deal with my problems on my own’) (Table 1).

This pattern was consistent for both women and men, although men reported slightly lower mean scores across all items compared to women. The lowest scores were observed for organisational barriers, such as ‘There were problems with my health insurance’ or ‘There were problems with things like transportation or time.’.

Participants with positive screening for depressive symptoms (68.9 %), anxiety symptoms (66.8 %), or low mental health-related quality of life (82.3 %) were more likely to report unmet need for professional help than their counterparts. These groups also showed significantly higher mean scores across almost all barrier domains (Table 2).

For participants with problematic substance use, no significant difference was found in the proportion reporting unmet need (59.4 % vs. 42.5 %). However, they more frequently reported fears related to the psychotherapeutic setting (e. g. ‘I was afraid the therapist might admit me to a psychiatric hospital against my will.’) and fear about public stigmatisation (e. g. ‘I was worried that others would think poorly of me if I started psychotherapy.’)

In sex-stratified analyses, women with depressive or anxiety symptoms, or with low mental health-related quality of life, consistently reported significantly higher scores across all five barrier domains compared with women without such conditions. Among women with problematic substance use, both internalized help-seeking stigma and fear of the psychotherapeutic setting were significantly more pronounced than among those without problematic use. Among men, however, only fear of the psychotherapeutic setting was consistently elevated across group comparisons compared to those without problematic use.

Associations between individual and regional factors and the likelihood of reporting unmet need for professional help were analysed using a multilevel model (Table 3). The findings indicate that differences in unmet need were almost entirely explained by individual-level characteristics. In the null model (i. e. a model without explanatory variables), the intraclass correlation coefficient (ICC) was below 0.01, suggesting that nearly all variance was attributable to the individual level, while differences between districts were negligible. None of the regional characteristics included in the analysis were significantly associated with unmet need.

Participants with depressive or anxiety symptoms, as well as those with low mental health-related quality of life, more frequently reported an unmet need. Individuals with sufficient health literacy were less likely to report unmet need compared to those with inadequate health literacy.

At the individual level, women were more than twice as likely as men to report unmet need for professional mental health care. Higher subjective social status was associated with a lower likelihood of reporting unmet need.

## 4. Discussion

The aims of the study were to identify barriers to accessing professional mental health care among young adults in Germany, to examine differences in help-seeking behaviour according to elevated care needs, and to analyse the associations with individual and regional characteristics. Overall, 42.6 % of the respondents reported that they never sought professional help despite perceiving a need or being advised to do so. The most prominent barriers related to problem denial (e. g. difficulties recognising one’s own need for help) and internalized help-seeking stigma, including the belief that one should cope alone and feelings of shame regarding one’s own mental distress. These findings are consistent with previous studies in Germany that identified help-seeking stigma and problem denial as central barriers in clinical samples [17, 32].

International studies likewise emphasise that internalised stigma and shame in dealing with mental health problems represent major barriers to help-seeking [15, 18]. In contrast, studies from the United States frequently report financial and organisational barriers, such as gaps in insurance coverage. The present findings suggest that such barriers are less prominent in the German context. However, a lower subjective social status was significantly associated with reporting unmet need, indicating that young adults’ perceived social and economic resources influence their help-seeking behaviour. No consistent association was found between health literacy and the perception of unmet need, although participants with sufficient health literacy were less likely to report unmet need. This aligns with current evidence suggesting that mental health knowledge correlates with more favourable attitudes towards help-seeking, but its effects on intentions – and particularly on actual help-seeking behaviour – tend to be small [44]. Nevertheless, prior studies have highlighted the importance of sufficient health literacy for navigating the health care system [18] and identifying appropriate support options [15]. This underlines the need to address social inequalities and informational gaps regarding low-threshold mental health services.

A particular challenge exists for the group of participants showing indications of need for professional help (e. g. symptoms of depression or anxiety, or with low mental health-related quality of life). In multivariate analyses, respondents with depressive or anxiety symptoms had a significantly higher likelihood of reporting unmet need for care (odds ratios between 1.6 and 1.8). Conversely, better mental health-related quality of life was associated with a lower probability of unmet need (OR < 1). This finding is concerning, as individuals experiencing psychological distress and low mental health-related quality of life are at greater risk of developing mental disorders and could particularly benefit from early preventive measures or psychotherapeutic support.

Consistent with previous research [45], women were more likely than men to report unmet need for professional help. This may reflect both a greater willingness to acknowledge mental health problems and differences in coping strategies and communication about psychological distress [46]. Sex-stratified analyses showed that women with mental health problems (e. g. depressive or anxiety symptoms, low mental health-related quality of life) reported significantly higher levels across nearly all barrier domains compared to women without such symptoms. Among men, however, the pattern was less consistent: depending on the indicator, only selected barrier domains showed significant differences. Fear of the psychotherapeutic setting was the only domain consistently elevated among men with psychological symptoms. These results suggest potential gender-specific patterns in the perception and appraisal of barriers and warrant further investigation in future research.

Regional characteristics, such as mental health service density or socioeconomic deprivation, showed no significant association with unmet need. This suggests that regional differences between urban and rural districts accounted for only a minimal proportion of the total variance, and that perceived barriers are more likely to be related to individual-level factors. The findings indicate that increasing the density of care provision alone is not enough to reduce the barriers that hinder people from seeking professional help. Developing gender-sensitive informational and support interventions could be beneficial to accommodate differing needs and perceptions.

Overall, the findings highlight that, beyond structural resources, individual barriers must be addressed more directly. Interventions aiming at promoting health literacy, destigmatisation of mental health disorders, and low-threshold awareness campaigns about available services may help bridge the gap between need and utilisation. Preventive approaches should target early stages, aiming to improve problem recognition and perceived need for help, reduce internalised stigma and problem denial, strengthen help-seeking self-efficacy and action competence (e. g. concrete step and appointment planning), and enhance navigation skills and knowledge of access routes [44]. Future research should examine how individual mental health literacy and self-awareness and evaluation processes can be strengthened through universal and/or selective interventions, enabling informed and deliberate decisions about help-seeking, rather than decisions shaped by misperceptions or negative attributions.

## Limitations

Several limitations should be considered when interpreting the findings. First, the assessment of unmet need for professional help and perceived barriers was based on retrospective self-report, which may be subject to recall bias.

Although weighting procedures were applied to adjust for unequal participation probabilities, generalisability is limited because the sample included a disproportionately high proportion of women and individuals with higher educational attainment. The cross-sectional design allows only for conclusions to be drawn about associations between needs and perceived barriers, but not causal inferences. A longitudinal approach could shed more light on changes in care needs, help-seeking behaviour and the long-term effects of barriers.

Another limitation concerns the wording of the question on the need for professional help. Combining the aspects of ‘recommendation by others’ and ‘self-perceived need’ may blur different motivational contexts. Nevertheless, the question enabled the identification of individuals with either self- or externally perceived unmet need. Moreover, participants with indications of mental distress (e. g. symptoms of anxiety or depression) were more likely to report unmet need, suggesting that the question adequately captured those with psychological distress.

Furthermore, severely affected or clinically diagnosed cases were underrepresented in the present sample. For this group, structural barriers such as long waiting times or limited service availability may be more relevant than indicated by our findings. In addition, the analysis did not account for all possible forms of professional help, as the regional indicators referred exclusively to licensed psychotherapists within statutory health care. Private providers, low-threshold services or, digital health applications (DiGA), which could also be relevant, were not considered. Moreover, possible spillover effects between neighbouring districts were not captured, leaving potential regional interdependencies unobserved.

Despite these limitations, the study provides important insights into the relevance of individual barriers to access to psychotherapeutic care among young adults. It identifies subgroups particularly affected by unmet need and highlights where targeted strategies to improve access may be most effective.

Another limitation concerns the comparative analyses between male and female adults. In particular, the number of men with problematic substance use was low, which limits statistical precision for this subgroup. Post-hoc power analyses indicated that statistical power in some of these comparisons fell below the recommended threshold of 80 %, and results should therefore be interpreted as exploratory and with caution.

## Conclusion

This study demonstrates that a substantial proportion of young adults in Germany do not seek professional mental health care despite perceiving a need or being advised to seek help. Those particularly affected include individuals with symptoms of depression or anxiety, lower subjective social status, or low mental health-related quality of life. At the same time, results from this non-clinical, population-based cohort suggest that individual factors, particularly difficulties in recognising or acknowledging one’s own need for help, may play a greater role than regional service characteristics.

Sex-specific analyses suggest that the intensity of perceived barriers differs between men and women, with psychologically distressed women reporting overall higher levels of perceived barriers. Among men, fear of the psychotherapeutic setting appeared to be a particularly important factor for non-utilisation of professional help.

To improve access to psychotherapeutic care, structural interventions alone (e. g. increasing provider density) appear insufficient. Instead, low-threshold psychosocial interventions should be strengthened, including health literacy promotion, destigmatisation of mental disorders, and targeted information campaigns encouraging reflection on one’s own need for support. Preventive measures should be more strongly tailored to the life phase of young adulthood, where early and sex-sensitive approaches may help address needs more effectively and reduce gender disparities in access to care. In the long term, such strategies may not only improve help-seeking behaviour but also reduce the risk of chronic mental health problems.

## Figures and Tables

**Table 1:** Mean values and standard deviations for items on the Barriers to Seeking Psychotherapy Scale Scale (BAPS) and from the CIDI interview for participants with unmet need (n = 1301; n = 988 women; n = 313 men). Values range from 1 (not at all applicable) to 6 (very applicable).

Scale	Item	Women		Men		Total	
		M	(SD)	M	(SD)	M	(SD)
Fear of public stigma	I was worried that others would think poorly of me if I started psychotherapy.	2.47	(1.67)	2.33	(1.54)	2.44	(1.63)
	I was afraid that others would think I was crazy if they found out that I was in psychotherapy.	2.18	(1.64)	1.95	(1.46)	2.10	(1.58)
Fear of psychotherapeutic setting	I was afraid that the psychotherapist would admit me to a psychiatric hospital against my will.	2.18	(1.68)	1.76	(1.34)	2.03	(1.58)
	I didn’t think psychotherapy would work.	3.16	(1.70)	3.24	(1.74)	3.19	(1.72)
Problem denial	I thought my problems were not severe enough to seek psychotherapy.	4.57	(1.57)	4.40	(1.51)	4.51	(1.55)
	I thought I was just being silly.	3.98	(1.72)	3.51	(1.81)	3.81	(1.76)
Internalized help-seeking stigma	I thought I had to deal with my problems on my own.	4.48	(1.55)	4.21	(1.61)	4.38	(1.58)
	I was ashamed of my problems.	3.28	(1.80)	3.11	(1.87)	3.22	(1.83)
Practical implementation/organisation	I did not find any practitioners/therapist.	2.94	(1.94)	2.29	(1.64)	2.70	(1.87)
	I did not get an appointment.	2.59	(1.97)	1.90	(1.56)	2.34	(1.86)
	The waiting time was too long.	2.95	(2.07)	2.13	(1.75)	2.66	(2.00)
	There were problems with health insurance/insurance.	1.68	(1.31)	1.39	(1.00)	1.58	(1.22)
	I did not like the practitioner/therapist.	1.84	(1.49)	1.49	(1.21)	1.71	(1.41)
	There were problems with things like transportation or time.	1.91	(1.50)	1.62	(1.40)	1.81	(1.47)

M = mean, SD = standard deviation

**Table 2:** Group comparison of perceived barriers according to depressive symptoms, anxiety symptoms, substance use and mental health-related quality of life (n = 1,301, n = 988 women, n = 313 men). Value range from 1 (not applicable at all) to 6 (very applicable).

Scale	Women					Men					Total				
	Depressive symptoms (n = 357)		No depressive symptoms (n = 631)			Depressive symptoms (n = 123)		No depressive symptoms (n = 190)			Depressive symptoms (n=480		No depressive symptoms (n = 821)		
	M	(SD)	M	(SD)	P	M	(SD)	M	(SD)	P	M	(SD)	M	(SD)	P
Fear of public stigmatisation	**2.64**	**(1.71)**	**2.13**	**(1.41)**	0.007	**2.48**	**(1.64)**	**1.93**	**(1.17)**	0.023	**2.58**	**(1.69)**	**2.05**	**(1.33)**	<0.001
Fear of psycho-therapeutic setting	**3.15**	**(1.47)**	**2.33**	**(1.15)**	<0.001	**2.82**	**(1.23)**	**2.32**	**(1.12)**	0.012	**3.04**	**(2.26)**	**2.32**	**(1.10)**	<0.001
Problem denial	4.46	**(1.45)**	4.14	(1.52)	0.063	3.97	**(1.64)**	3.96	(1.31)	0.951	4.30	**(1.46)**	4.07	(1.40)	0.129
Internalized help-seeking stigma	**4.33**	**(1.34)**	**3.56**	**(1.45)**	<0.001	**4.20**	**(1.43)**	**3.34**	**(1.38)**	0.001	**4.29**	**(1.33)**	**3.47**	**(1.40)**	<0.001
Practical implementation/organisation	**2.56**	**(1.36)**	**2.20**	**(1.22)**	0.015	2.03	**(1.14)**	1.76	(1.06)	0.167	**2.38**	**(1.32)**	**2.04**	**(1.08)**	0.004
	**Anxiety symptoms** **(n=482)**		**No anxiety symptoms** **(n = 506)**			**Anxiety symptoms** **(n = 126)**		**No anxiety symptoms** **(n = 187)**			**Anxiety symptoms** **(n = 669)**		**No anxiety symptoms** **(n = 632)**		
	**M**	**(SD)**	**M**	**(SD)**	**P**	**M**	**(SD)**	**M**	**(SD)**	**P**	**M**	**(SD)**	**M**	**(SD)**	**P**
Fear of public stigmatisation	**2.62**	**(1.69)**	**2.03**	**(1.35)**	0.001	**2.44**	**(1.59)**	**1.96**	**(1.21)**	0.042	**2.57**	**(1.66)**	**1.99**	**(1.29)**	<0.001
Fear of psycho-therapeutic setting	**2.95**	**(1.45)**	**2.36**	**(1.16)**	<0.001	**2.98**	**(1.33)**	**2.22**	**(0.99)**	<0.001	**2.96**	**(1.41)**	**2.29**	**(1.09)**	<0.001
Problem denial	**4.54**	**(1.39)**	**3.97**	**(1.56)**	0.001	4.00	**(1.58)**	3.94	(1.35)	0.841	**4.39**	**(1.47)**	**3.96**	**(1.48)**	0.002
Internalized help-seeking stigma	**4.29**	**(1.40)**	**3.42**	**(1.39)**	<0.001	**4.02**	**(1.36)**	**3.45**	**(1.48)**	0.031	**4.22**	**(1.39)**	**3.43**	**(1.42)**	<0.001
Practical implementation/organisation	**2.66**	**(1.34)**	**2.01**	**(1.13)**	<0.001	**2.20**	**(1.10)**	**1.66**	**(1.05)**	0.003	**2.53**	**(1.29)**	**1.86**	**(1.11)**	<0.001
	**Problematic substance use** **(n = 29)**		**No substance use** **(n = 959)**			**Problematic substance use** **(1 = 13)**		**No substance use** **(n = 300)**			**Problematic substance use** **(n=42)**		**No substance use** **(n = 1,259)**		
	**M**	**(SD)**	**M**	**(SD)**	**p**	**M**	**(SD)**	**M**	**(SD)**	**P**	**M**	**(SD)**	**M**	**(SD)**	**p**
Fear of public stigmatisation	3.22	(1.80)	2.32	(1.55)	0.101	2.98	(1.63)	2.10	(1.36)	0.176	3.10	(1.69)	2.23	(1.48)	0.041
Fear of psycho-therapeutic setting	**3.86**	**(1.33)**	**2.64**	**(1.34)**	<0.001	**3.16**	**(0.81)**	**2.47**	**(1.19)**	0.009	**3.52**	**(1.15)**	**2.58**	**(1.29)**	<0.001
Problem denial	4.77	(1.31)	4.26	(1.50)	0.116	3.24	(1.83)	4.00	(1.41)	0.281	4.02	(1.74)	4.17	(1.48)	0.745
Internalized help-seeking stigma	**4.63**	**(1.23)**	**3.86**	**(1.46)**	0.030	4.49	**(1.35)**	3.62	(1.46)	0.051	**4.56**	**(1.27)**	**3.77**	**(1.46)**	0.005
Practical implementation/organisation	2.77	(1.74)	2.34	(1.27)	0.422	2.31	(1.47)	1.84	(1.07)	0.423	2.54	(1.61)	2.16	(1.23)	0.343
	**High psychological HRQoL** **(n = 798)**		**Low psychological HRQoL** **(n** = **190)**			**High psychological HRQoL** **(n = 248)**		**Low psychological HRQoL** **(n** = **65)**			**High psychological HRQoL** **(n = 1,046)**		**Low psychological HRQoL** **(n** = **255)**		
	**M**	**(SD)**	**M**	**(SD)**	**P**	**M**	**(SD)**	**M**	**(SD)**	**P**	**M**	**(SD)**	**M**	**(SD)**	**P**
Fear of public stigmatisation	**2.18**	**(1.46)**	**2.92**	(1.78)	0.006	2.12	(1.36)	2.21	(1.36)	0.728	**2.65**	**(1.71)**	**2.16**	**(1.42)**	0.018
Fear of psycho-therapeutic setting	**2.51**	**(1.28)**	**3.28**	(1.44)	<0.001	**2.39**	**(1.19)**	**2.86**	**(1.19)**	0.025	**3.11**	**(1.33)**	**2.46**	**(1.25)**	<0.001
Problem denial	**4.18**	**(1.51)**	**4.63**	(1.43)	0.016	4.00	(1.39)	3.84	(1.39)	0.612	4.33	(1.54)	4.12	(1.47)	0.239
Internalized help-seeking stigma	**3.66**	**(1.44)**	**4.72**	(1.21)	<0.001	4.53	**(1.44)**	4.09	(1.44)	0.098	**4.48**	**(1.45)**	**3.61**	**(1.44)**	<0.001
Practical implementation/organisation	**2.27**	**(1.28)**	**2.64**	(1.30)	0.039	1.83	**(1.11)**	1.96	(1.11)	0.577	2.37	(1.25)	2.12	(1.24)	0.077

M=mean value, SD=standard deviation, HRQoL=Health Related Quality of Life

Values in bold are statistically significant (p<0.05).

**Table 3:** Results of the multivariate analysis for predicting unmet needs for professional help (n = 3,051, n = 2,012 women, n = 1,039 men).

Predictor	OR	(95 % CI)	p
District level (N = 230)			
Socioeconomic deprivation (Ref.: 1 low)			
2	0.99	(0.66 – 1.51)	0.456
3	1.21	(0.81 – 1.80)	0.612
4	1.23	(0.77 – 1.95)	0.406
5 (high)	1.12	(0.65 – 1.91)	0.679
Eastern Germany (Ref.: Western Germany)	0.99	(0.67 – 1.47)	0.953
District type according to Eurostat (ref.: predominantly urban)			
Intermediate	0.92	(0.67 – 1.27)	0.276
Predominantly rural	0.67	(0.43 – 1.04)	0.600
Medical psychotherapy practice/10,000 inhabitants	0.91	(0.63 – 1.31)	0.656
Psychological-psychotherapeutic practice/10,000 inhabitants	1.04	(0.96 – 1.13)	0.311
Individual level (N = 3,051)			
Female gender (Ref.: male)	**2.21**	(1.63 – 3.01)	< 0.001
Age	1.05	(0.99 – 1.12)	0.097
Subjective social status	0.94	(0.87 – 0.99)	0.049
Mental health-related quality of life (Ref.: poor)			
Fair	**0.04**	(0.00 – 0.35)	0.005
Good	**0.02**	(0.00 – 0.15)	< 0.001
Very good	**0.01**	(0.00 – 0.07)	< 0.001
Excellent	**0.00**	(0.00 – 0.03)	< 0.001
Depressive symptoms (Ref.: negative screening, < 3)	**1.67**	(1.16 – 2.41)	0.004
Anxiety symptoms (Ref.: negative screening, < 3)	**1.71**	(1.71 – 1.25)	0.001
Problematic substance use (Ref.: negative)	1.81	(0.47 – 7.01)	0.373
Health literacy (Ref.: inadequate)			
Problematic	0.86	(0.62 – 1.18)	0.389
Sufficient	0.65	(0.43 – 0.98)	0.047
Excellent	1.10	(0.87 – 1.03)	0.683
ICC = 0.00, conditional R^2^ = 0.313			

OR = odds ratio, 95 % CI = 95% confidence interval, Ref. = reference category, EW = population, ICC = intraclass correlation coefficient. Values in bold are statistically significant (p < 0.05).
